# The Effect of Inclusions on a Medium Temperature Transformation Microstructure and Toughness of High-Strength HSLA Steel

**DOI:** 10.3390/ma14164424

**Published:** 2021-08-07

**Authors:** Yang Yang, Xian-Ming Zhao, Hao Li, Xiao-Yu Zhao, Huai-Bin Han

**Affiliations:** The State Key Laboratory of Rolling and Automation, Northeastern University, Shenyang 110819, China; yangyang.neu@outlook.com (Y.Y.); lihao960729@163.com (H.L.); sunny_zhao_xy@163.com (X.-Y.Z.); 15239718910@126.com (H.-B.H.)

**Keywords:** high-strength HSLA steel, in situ, MnS, toughness

## Abstract

In this study, the evolution of high-strength HSLA steel microstructure was studied using high-temperature laser confocal microscopy and SEM, TEM, and EPMA techniques. The effect of precipitates on grain boundary migration of austenite during high-temperature heating and the effect of inclusions in undercooled austenite on AF phase transformation were studied. The effect of multiphase microstructure on impact toughness was studied by Gleeble thermal simulation at 550, 600, and 650 °C. The results show that the austenite grain is refined by TiN pinning at high temperatures, and a large number of NbC and VCN are precipitated in ferrite for precipitation strengthening. The (Ti-Mn-O) + (Al + Si + Mn-O) + MnS composite inclusions with smaller sizes have a greater promoting effect on the nucleation of acicular ferrite than single-phase MnS. With a decrease in isothermal temperature, the content of acicular ferrite increases. When the isothermal temperature is 550 °C, an increase in the maximum impact toughness of acicular ferrite with large-angle grain boundary is clearly observable.

## 1. Introduction

A current trend in the rapid development of the automobile industry is high-strength and lightweight materials [1,2]; therefore, the automobile industry has established higher requirements for the material properties of automobile parts [3]. High-Strength Low-Alloy steel (HSLA) has the advantages of fewer generation and manufacturing links, no heat treatment process, low generation cost, good environmental protection performance, and excellent mechanical properties; therefore, it has attracted the attention of many studies [4,5].

Microstructure and mechanical properties of materials are closely related. In the traditional metallurgical field, the influence of inclusions on steel is complex; it is well known that large nonmetallic inclusions are harmful to the mechanical properties of steel because they act as nucleation sites for pores and cracks during deformation and service, thereby becoming the source of cracks [6]. However, in HLSA steel, a large number of fine carbonitride precipitates play an important role in grain refinement and acicular ferrite (AF) transformation. In the field of welding, several studies have confirmed that AF structure is important for improving mechanical properties and impact toughness [7,8,9]. For studies on nonmetallic inclusions, the main experimental objects are oxides [10,11] and Ti-rich inclusions [12,13] such as Ti_2_O_3_, TiN, and MnS [14,15,16,17]. In a study by J M Dowling [18] et al., it was indicated that any inclusions surface can assist ferrite nucleation via simple heterogeneous nucleation The main mechanisms of AF nucleation are minimum mismatch mechanism [19], Mn-poor region mechanism [12,20], stress–strain energy mechanism, and inert interface energy mechanism [21]. It is widely accepted that AF refines grains, which improve mechanical properties. When a material receives external load impact, crack propagation deflects when it encounters AF, which consumes energy. The phase transformation process can be observed better by using updated equipment such as a high-temperature in situ confocal microscope [22,23,24,25]. Undercooled austenite transforms into polygonal ferrite (PF) and acicular ferrite (AF), granular bainite (GB) and lath bainite (LB), and Martensite (M) under different cooling conditions; however, most studies have focused on the welding thermal cycle. Complex microstructures can be obtained by the phase transformation of different cooling paths at an intermediate temperature. There are few studies [4,9,21] on the isothermal transformation process at an intermediate temperature and the influence of different multiphase structures on the impact toughness. In a study by Li [13] et al., it was shown that small inclusions of 2–4 microns increased the possibility of AF nucleation. Additionally, we concluded that larger MnS can also be used as a matrix for AF nucleation.

In this study, a suitable cooling rate range of acicular ferrite was determined by continuous cooling transformation experiment. We studied the evolution of high-strength HSLA steel microstructure during isothermal heat treatment at medium and high temperatures (650, 600, and 550 °C) by Gleeble 3800 and VL2000 DX, as well as the effect of different types of precipitates on the nucleation of acicular ferrite. In addition, we calculated the precipitation behavior of precipitates by thermodynamics, and investigated the effect of different complex-phase ratios on the impact toughness. Our study aimed to investigate the influence of inclusions on the medium temperature transformation, microstructure, and toughness of high-strength steel to find out the appropriate process to improve the material properties.

## 2. Materials and Methods

The experimental steel was an ingot melted in a vacuum induction furnace. The chemical composition (mass fraction, %) of the experimental steel was 0.25C-0.38Si-2.0Mn-0.05S-0.015Al-0.005NB-0.12V-0.015Ti, and Fe was balanced.

In order to obtain the cooling rate range suitable for acicular ferrite microstructure, the ingot was processed in a cylinder (diameter of 3 mm and a length of 10 mm) with a DIL 805 A/D series quenching dilatometers (TA Instruments, New Castle, DE, USA) (5 °C/s heating rate up to 1200 °C, and holding for 5 min). Then, the ingot was cooled to room temperature at cooling rates of 0.1, 0.5, and 1 °C/s, respectively.

The nucleation and transformation process of the intermediate temperature phase transformation microstructure was studied by isothermal heat treatment. The steel sample was processed in a cylindrical sample with a diameter of 7.5 mm and a height of 3 mm, and the upper surface of the sample was ground and polished. The sample was placed in the crucible of the heating furnace, and then helium gas was vacuum pumped in, for protection to prevent high-temperature oxidation of the sample. The high-temperature observations of the experimental steel were carried out by a VL2000DX high temperature laser confocal microscope (Yonekura Mfg. Co., Ltd., Osaka, Janpan), and the heating rate was raised to 1200 °C at 10 °C/s for 5 min. Then, the cooling rates at 3 °C/s were reduced to 550, 600, and 650 °C and held for 10 min. Finally, the sample was cooled to room temperature at a rate of 3 °C/s.

The steel sample was processed into a block sample of 11 × 11 × 60 mm^3^, and the heat treatment experiment was carried out on a Gleeble 3800 thermal simulator (Dynamic Systems Inc., New York, NY, USA), according to the same heat treatment process of high-temperature laser confocal. After heat treatment, the sample was cut into a 10 × 10 × 55 mm^3^ v-notched impact sample, and the room-temperature impact test was carried out on the impact testing machine (ZBC2452-C, SANS, Shandong, China). The average value of each process test was taken five times.

The experimental samples of the phase change instrument were cut into two halves along the center line of the thermocouple weld, and the metallographic samples were prepared by mechanical polishing with a diamond polishing paste. The upper surface of the sample in the high-temperature laser confocal heat treatment experiment was directly corroded using 4% nitric acid alcohol solution, and then cleaned and dried with acetone solution. The microstructure was observed by a Leica optical microscope. Twenty field-of-view positions were randomly selected for observation, and then IPP software (Media Cybernetics, Inc., Rockville, MD, USA) counted and organized the statistical content. The morphology and composition of inclusions were analyzed by scanning electron microscopy (Zeiss ULTRA 55, Oberkohen, Germany) and energy spectrum analysis. The samples for EBSD analysis were electropolishing in 90% ethanol and 10% perchloric acid. The EBSD analysis was carried out with a scanning step size of 0.3 um, and data was processed using Channel 5 software. In addition, the impact fracture morphology and the crack morphology perpendicular to the fracture were observed by SEM. The scanning spectrum of element distribution on the surface of inclusion was analyzed by EPMA (JEOL JXA 8530F, Toyoshima, Tokyo, Japan). The microstructure under different processes was observed by transmission electron microscope (Tecnai G2F20, FEI, Hillsboro, OR, USA) and an energy spectrometer. Prior to this, the sample was polished and etched in 8% perchloric acid alcohol at −30 °C. The CCT and TTT curves of the experimental steel were simulated using JMatPro software (JMatPro software, Guildford, UK). The precipitation behavior of precipitates in experimental steel was calculated by TCFE9 database in Thermo-Calc software (Thermo-Calc Software AB, Solna, Sweden).

## 3. Results

**Figure 1:**
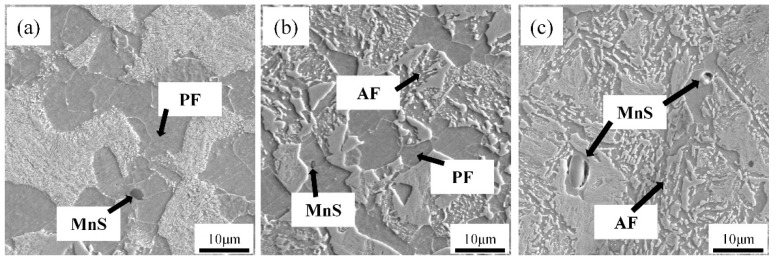
Optical micrographs of steel, cooled continuously at different cooling rates following austenitization at 1523 K for 30 min: (**a**) 0.1 °C/s; (**b**) 0.°C/s; (**c**) 1 °C/s.

Figure 1 shows the microstructure of the experimental steel during continuous cooling transformation at cooling rates of 0.1, 0.5, and 1 °C/s. When the cooling rate is 0.1 °C/s, the sample consists of massive PF and pearlite. When the cooling rate increases to 0.5 °C/s, the microstructure is mainly composed of AF and a small amount of PF, and the PF size decreases before the intersection. When the cooling rate is 1 °C/s, the microstructure is mainly fine acicular ferrite and bainite, with uniform microstructure; PF was not found.

**Figure 2:**
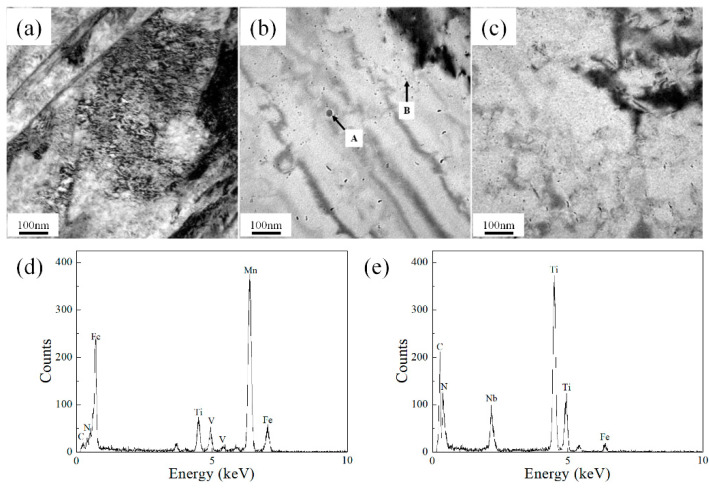
TEM micrographs: (**a**) AF morphology; (**b**) precipitation morphology at a cooling rate of 0.1 °C/s; (**c**) precipitation morphology at a cooling rate of 1 °C/s; (**d**) EDS result at position A; (**e**) EDS result at position B.

Figure 2 shows the microstructure of continuous cooling. Figure 2a shows that acicular ferrite presents a substructure similar to that of lath or acicular band. A large number of high-density dislocations were found in acicular ferrite. Figure 2b,c show the precipitation of ferrite at cooling rates of 0.1 °C/s and 1 °C/s, respectively. Through the energy spectrum measurements of precipitates with different sizes, the NbCN precipitates with a large size of approximately 38 nm at position A and VCN precipitates at position B can be obtained.

**Figure 3:**
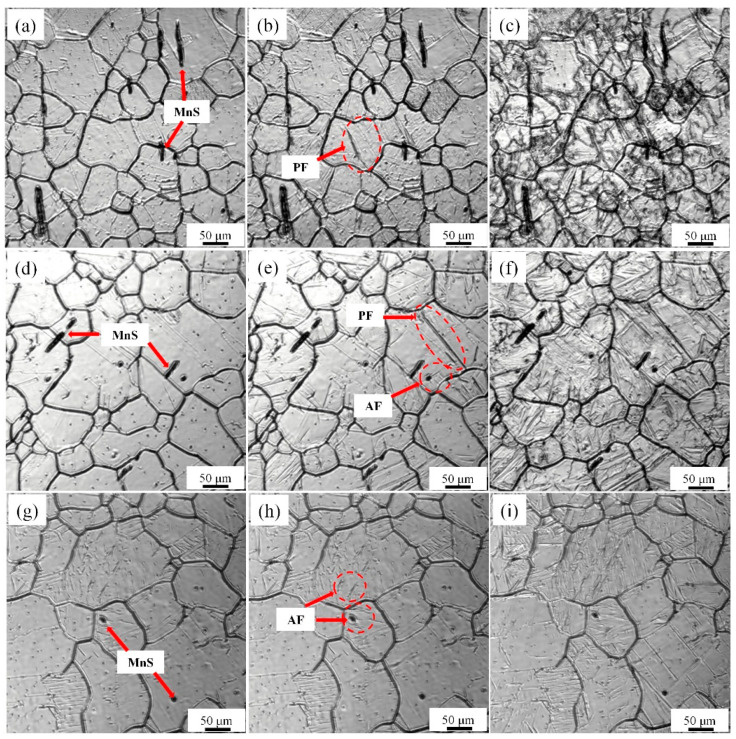
In situ observation of phase transformation microstructure at different holding temperatures: (**a**–**c**) 650 °C; (**d**–**f**) 600 °C; (**g**–**i**) 550 °C.

Figure 3 shows the in situ observations of tissues during different isothermal heat treatment processes. It can be observed from the diagram that there are two kinds of precipitates in the sample: (1) one precipitate has a large number of nm precipitation, and the TiN distribution displays a very uniform dispersion and (2) the other precipitate is a short rod-like MnS with a length of approximately 3 to 50 microns. Both precipitates can effectively limit the movement of austenite grain boundaries during heating. It can be observed in Figure 3b that when the isothermal temperature is 650 °C, austenite first transforms into PF, and then the remaining untransformed austenite continues to undergo pearlite transformation, as shown in Figure 2c. When the isothermal temperature drops to 600 °C, the transformation of austenite to PF and AF occurs. PF nucleation on the grain boundary and AF nucleation on the inclusions in the austenite grain have no P transformation. The normal growth of AF is random. When the isothermal temperature continues to drop to 550 °C, almost only austenite transforms to acicular ferrite during the isothermal process, and AF in different directions collide.

**Figure 4:**
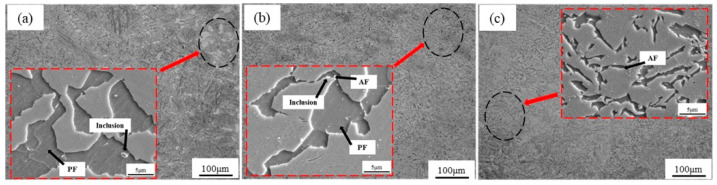
Optical micrographs of steels transformed at different temperatures for 10 min following austenitization at 1200 °C for 5 min: (**a**) 650 °C; (**b**) 600 °C; (**c**) 550 °C (the image in the red box is a partially enlarged SEM image in the position of the arrow).

As shown in Figure 4, after isothermal heat treatments at 650, 600, and 550 °C, the microstructure is mainly composed of PF + B, PF + AF + B, and AF + B. PF is equiaxed, when the isothermal temperature decreases from 650 to 600 °C, the average width decreases from approximately 16.9 to approximately 13.5 μm. When the cooling rate further decreases to 550 °C, PF disappears, and for almost all AF, the average length is approximately 8.6 microns, and the average width is approximately 2.3 microns.

**Figure 5:**
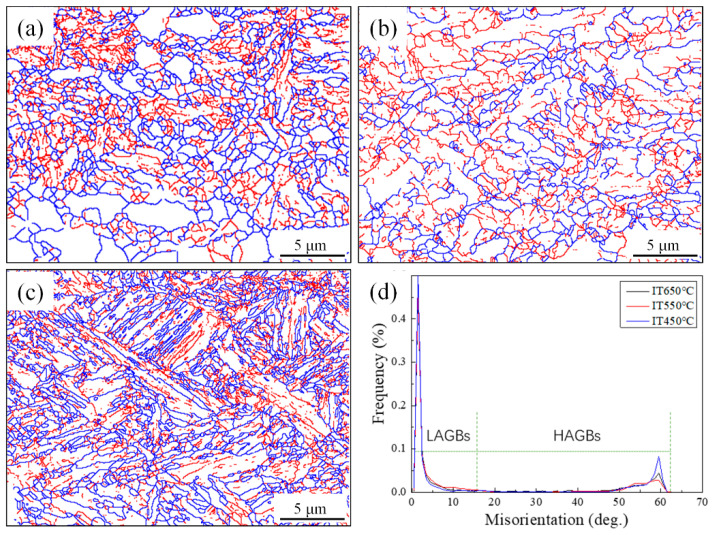
EBSD analysis of microstructure corresponding to isothermal temperatures of (**a**) 650 °C; (**b**) 600 °C; (**c**) 550 °C; (**d**) grain boundary misorientation distribution (blue lines denote the angles >15° and red lines denote the angle between 2 and 15°).

Figure 5 shows the EBSD grain boundary orientation difference of the microstructure after different isothermal heat treatments. It is well known that the grain boundary angles of bainite, polygonal ferrite, and ferrite laths are less than 15°; therefore, we used the 15° boundary to study the grain boundary. The blue line represents the angle >15° and the red line represents the angle between 2 and 15°. The orientation difference between acicular ferrite and adjacent lath ferrite is large. By measuring the average effective grain size, the grain size is from 2.6 to 8.6 μm. At 650, 600, and 550 °C, the proportions of the large-angle grain boundaries are 24.24%, 26.29%, and 31.36%, respectively, which is closely related to the content of acicular ferrite.

**Figure 6:**
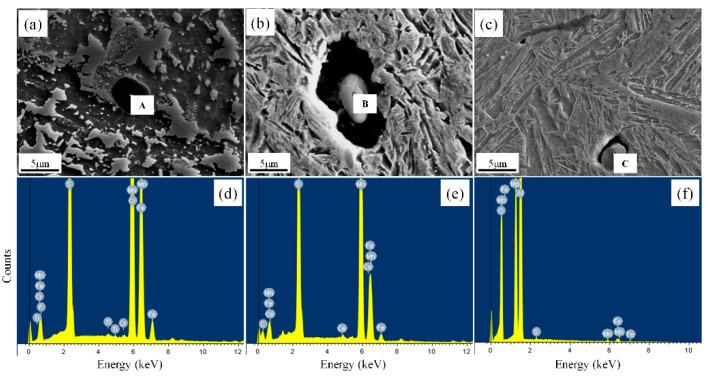
SEM micrographs and EDX spectra of typical nonmetallic inclusions: (**a**) MnS-Ti_2_O_3_; (**b**) MnS; (**c**) MnS-Al_2_O_3_; (**d**) EDX spectrum at position A; (**e**) EDX spectrum at position B; (**f**) EDX spectrum at position C.

Figure 6 shows the SEM micrographs and EDX spectra of typical nonmetallic inclusions in the experimental steel. The EDX spectra show that the inclusions at position A are manganese-titanium oxide composite type, the inclusions at position B are manganese type, and the inclusions at position C are manganese-alumina composite type. The existing experimental conditions cannot exclude the carbon, chromium, and iron shown in the EDX spectra as the matrix of steel. The precipitated nonmetallic inclusions are spherical or elliptical, and range in size from approximately 3.1 to 5.3 μm; some inclusions are rolled strip MnS and are approximately 13.5 μm in size.

**Figure 7:**
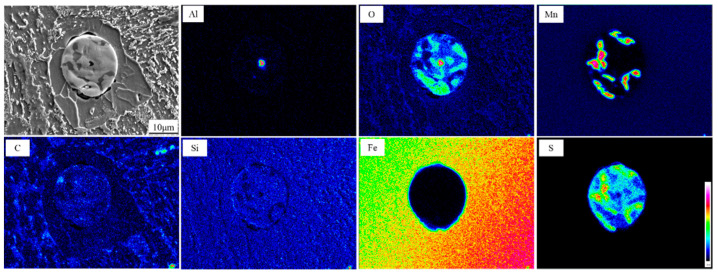
EPMA analysis of one typical inclusion particle effective for AF nucleation in steel with chemical maps of different elements.

Figure 7 shows the electron probe surface scanning analysis of typical nonmetallic inclusions in the experimental steel. The results show that the composite nonmetallic inclusions act as nucleation particles in fine ferrite. The shapes of the inclusions are spherical, and the sizes are approximately 18 μm. The central part of each inclusion is round Al_2_O_3_, and the size is 1.6 μm. Al_2_O_3_ is surrounded by MnS. On the basis of these results, it can be inferred that the reaction formulae of inclusion formations are: Ti + O = Ti_2_O_3_, Si + O = SiO_2_, Al + O = Al_2_O_3_, Mn + O = MnO, and Mn + S = MnS. Therefore, a composite inclusion in the form of (Ti-Mn-O) + (Al + Si + Mn-O) + MnS was finally formed. Since manganese is a stable austenite element, the formation of MnS limits the Mn element in the matrix to a certain range, forming a poor Mn band. This also promotes the transformation from austenite instability to ferrite.

**Figure 8:**
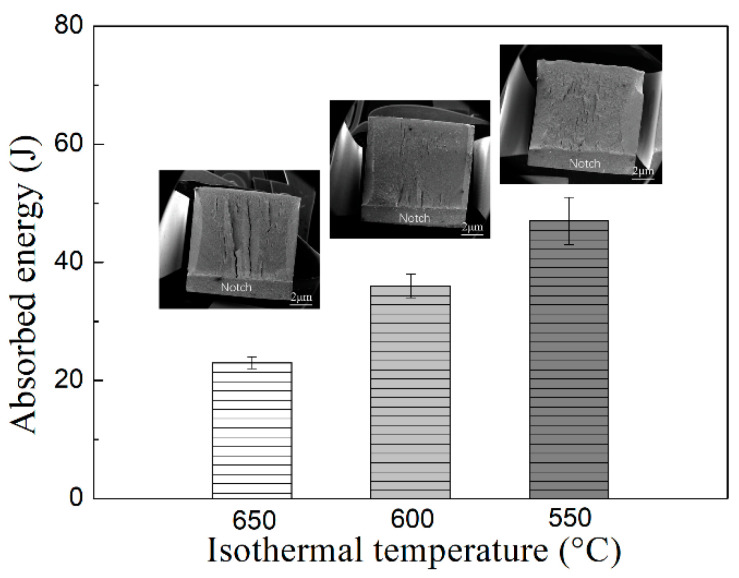
The average CVN impact test results at three different isothermal temperatures and the corresponding macroscopic fractures that are obtained (error bar is the SD).

**Figure 9:**
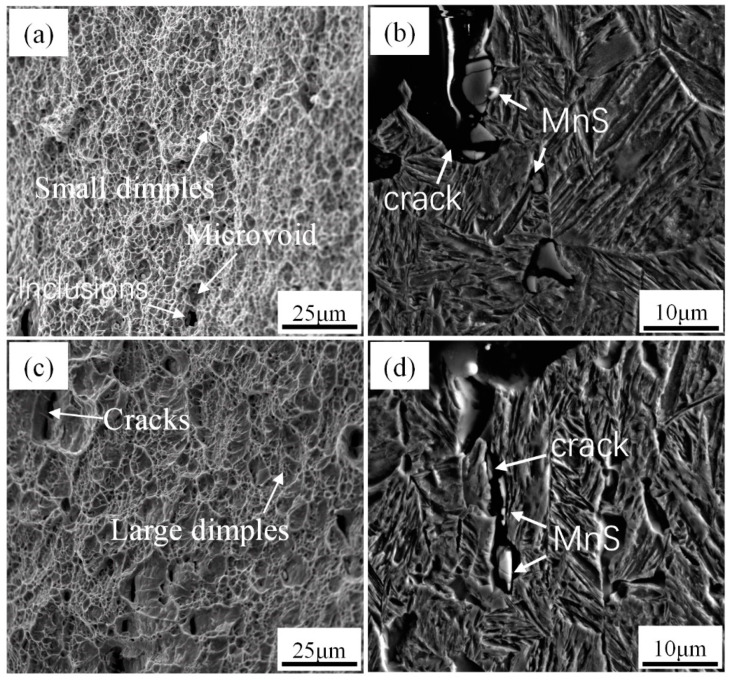
SEM micrographs of fracture surfaces and corresponding micrographs perpendicular to the fracture surface of (**a**,**b**) 550 °C and (**c**,**d**) 600 °C isothermal samples.

Figure 8 shows the impact energy and corresponding macroscopic fracture morphology of CVN samples at different isothermal temperatures. The average impact energy is 23 ± 1 J after isothermal heat treatment at 650 °C. With a decrease in isothermal temperature from 650 to 600 °C, the toughness increases by approximately 56% (36 ± 2 J). When the isothermal temperature continues to decrease to 550 °C, the toughness increases by approximately 104% (47 ± 4 J). The characteristic of quasi-cleavage fracture can be observed in the fracture surface, as shown in Figure 8. The number of cracks increases, and the surface becomes flatter with decreasing isothermal temperature; additionally, the area of ductile fracture zone increases. In order to study the improvement effect of acicular ferrite on impact properties in detail, the fracture morphology and SEM micrographs perpendicular to the fracture morphology of the samples at 600 and 550 °C are shown in Figure 9. Under the isothermal process at 600 °C, the microstructure is composed of polygonal ferrite, acicular ferrite, and bainite. The ferrite and bainite phases coordinately deform during plastic deformation, forming a certain number of micropores during necking fracture, but the number of micropores is small and the size is small, as shown in Figure 9.

## 4. Discussion

### 4.1. Effects of Precipitate Behavior on Microstructure Refinement

**Figure 10:**
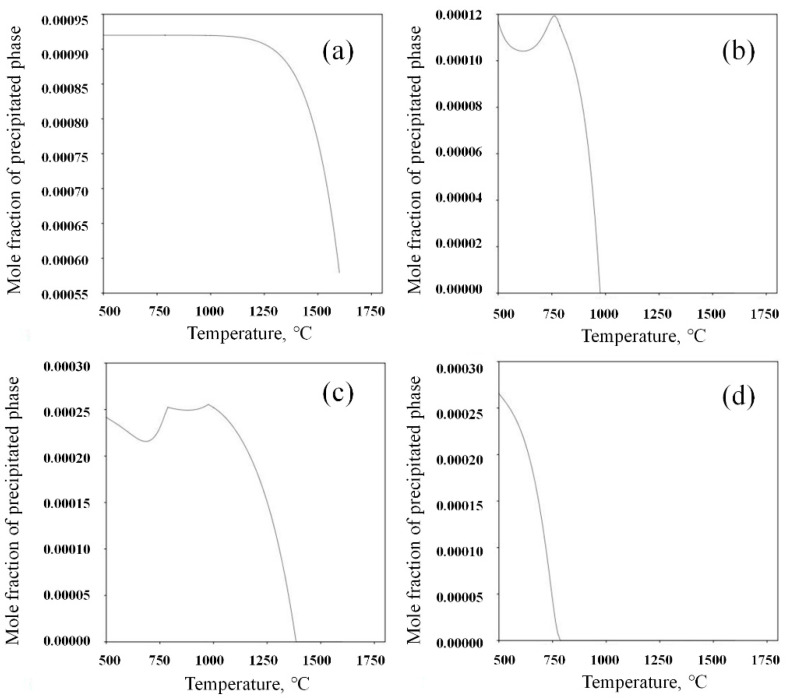
Calculated equilibrium mole fractions of precipitated phases in steels: (**a**) MnS; (**b**) NbCN; (**c**) TiN; (**d**) VCN.

The precipitates (e.g., MnS, TiN, NbCN, VCN) precipitate from austenite during the solidification of molten steel with changes in the cooling temperature. The results of the volume fraction of precipitates with changes in temperature were calculated with Thermal-Calc, as shown in Figure 10. The precipitation temperatures of the main precipitates in the experimental steel were 1534 °C for MnS, 1306 °C for TiN, 983 °C for NbCN, and 780 °C for VCN. The precipitation curve of MnS is parabolic, and shows that the precipitation rate slowed down with a decrease in temperature. NbC [26] reached the maximum precipitation at approximately 800 °C, and then the precipitation decreased with a change in solubility. A small trough peak appears at 700 °C. The maximum precipitation rate of TiN [27] is in the temperature range 1000–1400 °C. At 1000 °C, the precipitation amount is 0.00025 mole fraction. After the precipitation demonstrates a wavy pattern, the curve reaches the peak. The precipitation curve of VCN [28] is parabolic and shows that precipitation began at approximately 800 °C. The maximum precipitation mole fraction is 0.0026. Therefore, when the experimental steel is heated above 1150 °C, the main precipitates in austenite are MnS and TiN. As shown in Figure 2 and Figure 3, the size of TiN is in nanometers and MnS is in microns. It can be observed that the average size of nm precipitates is approximately 46 nm, and a large number of nm precipitates effectively pin the migration of austenite grain boundaries, thereby limiting the outward expansion trend of austenite grain boundaries. During the heating process of the sample, with the diffusion of solute elements and an increase in solubility, the precipitates disappear and dissolve into the matrix. Interestingly, it can be seen that there is a large difference in the size of austenite in the positions of the precipitates. The average austenite grain size without precipitates is larger than that with precipitates. According to the in situ observations of austenite growth in Figure 3, the mass fractions of MnS and TiN at 1100 °C were 0.092% and 0.024%, respectively. These insoluble precipitated particles effectively pinned the grain boundaries and refined the grain size to 70 microns.

**Figure 11:**
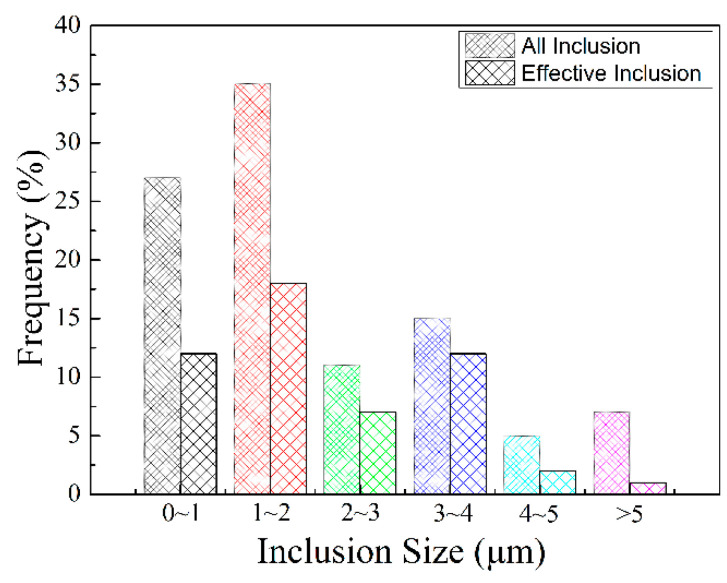
Relative ferrite nucleant size distribution superimposed on the size distributions of all inclusions.

Through the statistical analysis of the size distribution, the relative AF nucleating agent in steel is superimposed on the size distribution of all inclusions, as shown in Figure 11. When the sample is cooled, the elements, after diffusion and solid solution, reaggregate to form new inclusions. The basis of inclusion nucleation can be revealed by the Ostwald process. Ostwald quantified precisely that small particles have a higher solubility than larger particles; with decreasing temperature, the surface energy increases and the solubility of the particles increases. The largest number of inclusions in the steel is 1~2 microns, accounting for 35.1%; the second largest number of inclusions in the steel is 0~1 micron, accounting for 26.8%; the least number of inclusions are 4~5 microns and greater than 5 microns accounted for 5.4% and 6.8%, respectively. It can be concluded from the statistical analysis that when the size of inclusions is in the range of 3–4 microns, it accounts for 15.3% of the total. Among them, 12.7% of the inclusions become the nucleation particles of acicular ferrite, and the nucleation rate reaches 83%. According to the EDX spectrum results of inclusions in Figure 6, it can be seen that it is not easy for pure MnS inclusions to form AF nucleation particles. Inclusions with multiple types of inclusions, such as MnS-Ti_2_O_3_, MnS-Al_2_O_3_, and (Ti-Mn-O) + (Al + Si + Mn-O) + MnS with more complex structures shown in Figure 8, can better promote AF nucleation. It is inferred that this is the result of multiple mechanisms that promote ferrite nucleation. The lattice mismatch between TiN and ferrite is small, which reduces the activation energy barrier of ferrite nucleation. The results obtained from the J. M. Dowling study show that any inclusion surface can help ferrite nucleation through simple heterogeneous nucleation [18]. In the study of inclusions promoting nucleation with large size, it was found in the EPMA element surface scanning results that due to the formation of MnS, Mn elements in the local area formed a poor area. It is well known that Mn is the element of stable austenite, and the area lacking Mn also increases the chemical driving force of ferrite nucleation; therefore, it is more prone to ferrite transformation.

### 4.2. Effects of Microstructure Evolution of Different Isothermal Heat Treatments on Toughness

**Figure 12:**
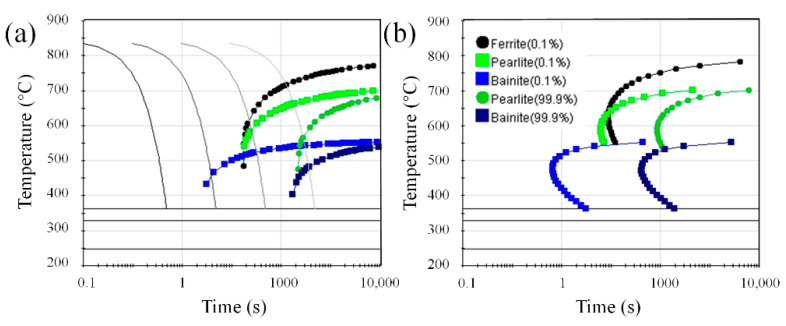
Phase transformation curves: (**a**) CCT; (**b**) TTT, with Jmat-Pro calculated steel.

**Figure 13:**
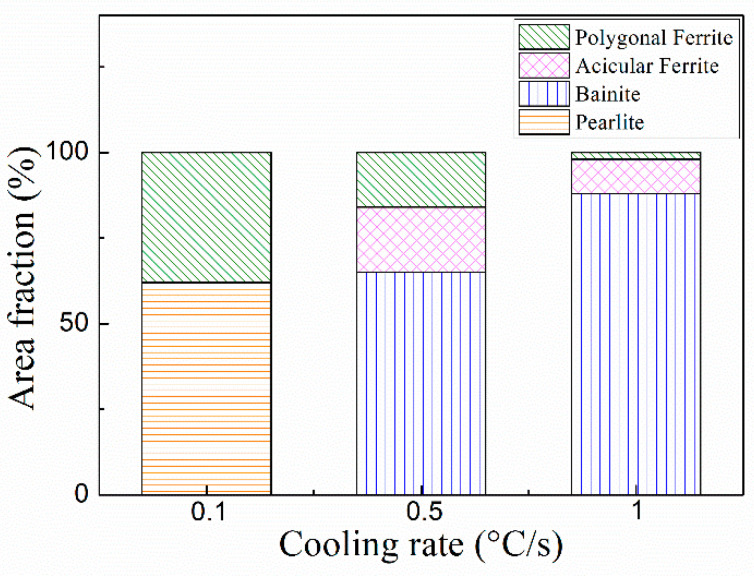
Area fraction of microstructural constituent at different cooling rates.

The continuous cooling transformation curve (CCT) and isothermal transformation curve (TTT) of austenite of the experimental steel are shown in Figure 12. The results show that the temperature range suitable for ferrite transformation is 0.1–1 °C/s for continuous cooling transformation. With an increase in cooling rate from 0.1 to 1 °C/s, pearlite and ferrite transformation occurs in austenite, resulting in the microstructure of P, PF, AF and B, which is consistent with the microstructure in Figure 1. According to the results of area fraction of microstructure components under different cooling rates, as shown in Figure 13, the microstructure changes from P + PF volume fractions of 62% and 38% to AF + PF + B volume fractions of 2%, 10%, 88%, respectively. Austenite preferentially transformed into ferrite at 650 °C, and then the untransformed austenite continued to diffuse into pearlite over time. With a decrease in isothermal temperature, it is expected that PF transformation decreases, acicular ferrite transformation increases, and bainite occurs. The microstructure of the samples at different isothermal temperatures is shown in Figure 4, and the enlarged SEM image of the position is shown in the circle in the figure. The higher isothermal temperature promotes the diffusion reaction of polygonal ferrite. As a result of this decrease in isothermal temperature, the volume fraction of polygonal ferrite decreases gradually, and the content of acicular ferrite increases significantly. This result is also consistent with the simulation prediction results.

The microstructure was further analyzed by TEM. As shown in Figure 2, polygonal ferrite and acicular ferrite have different dislocation densities. The acicular ferrite has a slender lath with higher dislocation density and a random orientation of interwoven morphology. The dislocation density in polygonal ferrite is low and equiaxed. It can be seen from Figure 2b,c that the interphase precipitation was refined with an increase in cooling rate, which decreased from an average of approximately 21 to 12 nm. The critical interface solute concentration required for carbide precipitation had not yet been reached. This means that the extent and size of interphase precipitation can be controlled by controlled cooling.

**Figure 14:**
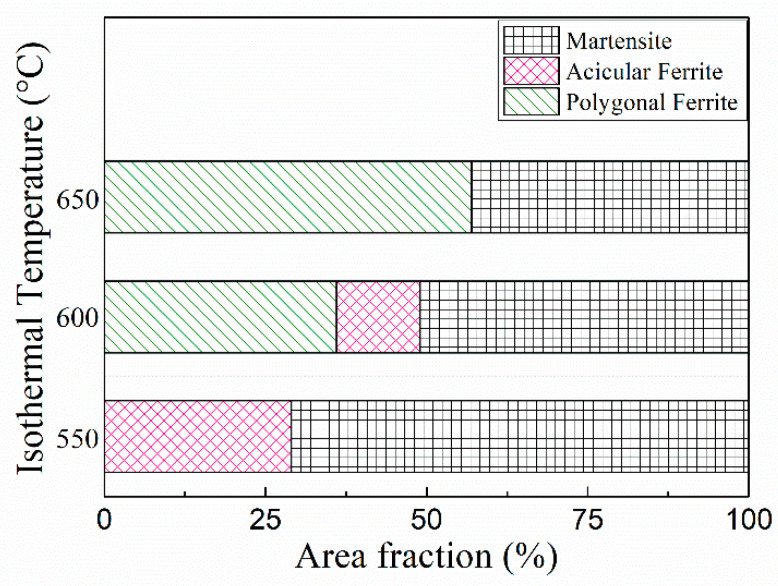
Area fraction of microstructural constituent at different isothermal temperatures.

During the cooling process, as the carbon atom diffuses from the ferrite into an austenite, the carbon atom is proliferated from the austenite that is converted from the ferrite, and the carbonaceous bone has increased stability. At the end of the isothermal process, the acicular ferrite transformation is completed. As the cooling rate in this experiment is small, the austenite continues to transform into bainite, and martensite transformation cannot occur. However, due to the existence of carbon-rich austenite, the final M/A structure is formed. According to the statistical results of area fraction of microstructure components at different isothermal temperatures in Figure 14, when the isothermal temperature is at 650 °C, the proportion of PF transformation is approximately 57%, and the remaining 43% is B. When the isothermal temperature decreases to 600 °C, the proportion of PF decreases approximately 36%, AF transformation is 13%, and the remaining 51% is B. When the isothermal temperature continues to decrease to 550 °C, almost no PF is found. The AF content continues to increase to 29%, and the remaining B accounts for approximately 71%.

When the isothermal temperature is 550 °C, the microstructure is composed of acicular ferrite and bainite. The acicular ferrite has a significant influence on impact toughness, because its fine grain size and the interlocking structure of large-angle boundary hinder the crack propagation. It can be seen from the EBSD crystal orientation diagram in Figure 5 that the proportion of large-angle grain boundary after the isothermal temperature decreases from 600 to 550 °C is 26.29% and 31.36%, respectively, and together with effective grain refinement led to increased toughness. When the specimen is deformed under an external load, the failure mainly originates from the microstructure, such as vacancy, dislocation, grain boundary, and micro crack. Inclusion MnS is an important cause of crack source [29]. Gao [30] et al. studied micro crack propagation and connectivity of ductile materials and showed that AF had higher requirements for crack initiation and propagation, and larger ductile fracture area. When the sample received plastic deformation, the cleavage crack was deflected by AF crystal orientation, so that the toughness improvement effect was greater than that of grain refinement.

## 5. Conclusions

In this study, the precipitation phase transformation microstructure of high-strength HSLA steel at an intermediate temperature and its influence on toughness were studied. The main conclusions are as follows:

(1) At a cooling rate of 0.1–1 continuous cooling, the austenite transforms into P + PF + AF + B. With an increase in cooling rate, the microstructure changes from 62% and 38% of P + PF volume fraction to 2%, 10%, and 88% of AF + PF + B volume fraction, respectively. The size of precipitates decreases.

(2) Different types of inclusions have different effects on the nucleation of acicular ferrite. Large-size single-phase MnS larger than 5 microns has a hysteresis effect on the nucleation of intragranular ferrite. In contrast, 3–4 micron small-size TiN inclusions and composite inclusions MnS-Al_2_O_3_ and MnS-Ti_2_O_3_, as well as (Ti-Mn-O) + (Al + Si + Mn-O) + MnS, have a stronger promoting effect on the nucleation of intragranular ferrite.

(3) With a decrease in isothermal temperature from 650 to 550 °C, the intermediate temperature phase transition product changed from 57% PF + 43% B to 29% AF + 71% B. The average impact energy is 23 ± 1 J after isothermal heat treatment at 650 °C. With a decrease in isothermal temperature from 650 to 600 °C, the toughness increases by approximately 56% (36 ± 2 J). When the isothermal temperature continues to decrease to 550 °C, the toughness increases by approximately 104% (47 ± 4 J). Both are quasi-cleavage fracture forms, and inclusion MnS is an important reason for the crack source. The maximum content of AF at 550 °C increases the proportion of large-angle grain boundaries, which together with grain refinement leads to improved toughness.

## Data Availability

Not applicable.
